# Performance of Four Respiratory Rate Counters to Support Community Health Workers to Detect the Symptoms of Pneumonia in Children in Low Resource Settings: A Prospective, Multicentre, Hospital-Based, Single-Blinded, Comparative Trial

**DOI:** 10.1016/j.eclinm.2019.05.013

**Published:** 2019-06-10

**Authors:** Kevin Baker, Tobias Alfvén, Akasiima Mucunguzi, Alexandra Wharton-Smith, Emily Dantzer, Tedila Habte, Lena Matata, Diana Nanyumba, Morris Okwir, Monica Posada, Anteneh Sebsibe, Jill Nicholson, Madeleine Marasciulo, Rasa Izadnegahdar, Max Petzold, Karin Källander

**Affiliations:** a Malaria Consortium, London, United Kingdom; b Department of Public Health Sciences, Karolinska Institutet, Stockholm, Sweden; c Sachs' Children and Youth Hospital, Stockholm, Sweden; d Malaria Consortium, Kampala, Uganda; e Malaria Consortium, Phnom Penh, Cambodia; f Malaria Consortium, Addis Ababa, Ethiopia; g Malaria Consortium, Juba, South Sudan; h Malaria Consortium, Raleigh, USA; i Bill & Melinda Gates Foundation, Seattle, USA; j Gothenburg University, Gothenburg, Sweden; k University of the Witwatersrand, Johannesburg, South Africa

**Keywords:** Childhood pneumonia, Low-income country, Diagnostic tools, Respiratory rate counting, Health worker performance

## Abstract

**Background:**

Pneumonia is one of the leading causes of death in children under-five globally. The current diagnostic criteria for pneumonia are based on increased respiratory rate (RR) or chest in-drawing in children with cough and/or difficulty breathing. Accurately counting RR is difficult for community health workers (CHWs). Current RR counting devices are frequently inadequate or unavailable. This study analysed the performance of improved RR timers for detection of pneumonia symptoms in low-resource settings.

**Methods:**

Four RR timers were evaluated on 454 children, aged from 0 to 59 months with cough and/or difficulty breathing, over three months, by CHWs in hospital settings in Cambodia, Ethiopia, South Sudan and Uganda. The devices were the Mark Two ARI timer (MK2 ARI), counting beads with ARI timer, Rrate Android phone and the Respirometer feature phone applications. Performance was evaluated for agreement with an automated RR reference standard (Masimo Root patient monitoring and connectivity platform with ISA CO_2_ capnography). This study is registered with ANZCTR [ACTRN12615000348550].

**Findings:**

While most CHWs managed to achieve a RR count with the four devices, the agreement was low for all; the mean difference of RR measurements from the reference standard for the four devices ranged from 0.5 (95% C.I. − 2.2 to 1.2) for the respirometer to 5.5 (95% C.I. 3.2 to 7.8) for Rrate. Performance was consistently lower for young infants (0 to < 2 months) than for older children (2 to ≤ 59 months). Agreement of RR classification into fast and normal breathing was moderate across all four devices, with Cohen's Kappa statistics ranging from 0.41 (SE 0.04) to 0.49 (SE 0.05).

**Interpretation:**

None of the four devices evaluated performed well based on agreement with the reference standard. The ARI timer currently recommended for use by CHWs should only be replaced by more expensive, equally performing, automated RR devices when aspects such as usability and duration of the device significantly improve the patient-provider experience.

**Funding:**

Bill & Melinda Gates Foundation [OPP1054367].

**Research in context**

**Evidence before this study**

We searched PubMed, the Cochrane Controlled Clinical Trials Register, and ClinicalTrials.gov database for relevant published articles and current trials assessing the accuracy, usability and acceptability of pneumonia diagnostic aids for use by frontline health workers in children under five. We used the search terms “pneumonia diagnostic aid” or “pneumonia diagnostic device” or “diagnostic” and “community health worker” or “frontline health worker” and “children under five” and “clinical trial” or “randomised control trial” or “study”. We limited the search to studies published from Jan 1, 1990 to Jan 1, 2017. We found no Cochrane systemic reviews or large scale randomised control trials of pneumonia diagnostic aids. We found a number of small scale studies of various pneumonia diagnostics aids for frontline health workers.

**Added value of this study**

To the best of our knowledge, our study is the first large, multi-centre trial evaluating the use of pneumonia diagnostic aids by community health workers in children under five. Our study, with its pragmatic design for resource poor settings, makes the results generalisable to other similar settings and populations.

**Implications of all the available evidence**

Although we did not see good agreement between any of the four devices tested and the reference standard, the findings of our study are consistent with other, smaller studies, which showed that accurately counting respiratory rate is difficult for community health workers. New, automated respiratory rate counters and other diagnostic tools are required if community health workers are to effectively detect the signs and symptoms of pneumonia in children under five, but their introduction should only be considered if they are shown to significantly improve the accuracy in diagnosis or the patient-provider encounter.

Alt-text: Unlabelled Box

## 1 Background

Pneumonia is the leading cause of post-neonatal death in children under-five years, accounting for an annual 944,000 deaths globally; 16% of all under-five mortality worldwide [1]. Sixty percent of these deaths occur in just 10 countries in South Asia and sub-Saharan Africa [2], many of which face significant challenges in provision of effective health care, diagnosis and treatment. Deaths from pneumonia in children result mostly from delayed presentation to appropriate care providers, inappropriate treatment or presumption the symptoms are due to malaria [3]. While caregivers may recognise rapid breathing in a coughing child, it does not always prompt them to seek care, resulting in delays and subsequent development of severe disease [3], [4], [5]. Children with severe pneumonia often have chest in-drawing, stridor and wheezing; symptoms which some health care workers are not able to adequately recognise and subsequently treat or refer for necessary antibiotic treatment and oxygen therapy [6].

Diagnosis of pneumonia by community health workers (CHWs), currently includes checking for danger and referral signs and subsequently counting the number of breaths for 60 s in children with history of cough and/or difficulty breathing, to assess whether the respiratory rate (RR) is higher than the normal parameters for a child of that age, as defined by the World Health Organisation (WHO) [7]. In the early 1990's, the WHO and the United Nations Children's Fund (UNICEF) issued a call for the development of a one-minute acute respiratory infection (ARI) timer to assist CHWs in measuring the length of time to count the RR in children. However, even with the deployment of the ARI timer counting RR continues to prove challenging for trained health workers and misclassification of the observed RR remains high [4], [8], [9], [10], [11], partly due to difficulty in trying not to lose count and also device characteristics such as a ticking sound every second [12].

Integrated community case management (iCCM) is an approach recommended by WHO, UNICEF and partners where CHWs are trained to identify and treat symptoms of pneumonia, malaria, and diarrhoea in children under-five years, as well as to detect and refer malnutrition and severely ill children to the nearest health facility. Evidence from African countries shows that CHWs, if properly trained and equipped, can potentially reduce child deaths from malaria, pneumonia and diarrhoea by up to 60% through the delivery of iCCM [13], [14], [15]. The identification and evaluation of new diagnostic tools for improved classification of pneumonia at the community level ranked fifth of 20 research priorities identified by a panel of global experts in 2014, and second in terms of importance and potential impact [16].

Wider use of improved RR diagnostic aids for pneumonia in low-resource settings are expected to contribute to more accurate detection and classification of pneumonia, and more appropriate use of antibiotics [17], [18], [19], [20]. This study aimed to assess the accuracy of four different RR counting aids to assess RR by frontline health workers in Cambodia in Southeast Asia, and Ethiopia, South Sudan and Uganda in sub-Saharan Africa.

## 2 Methods

**Fig. 1:**
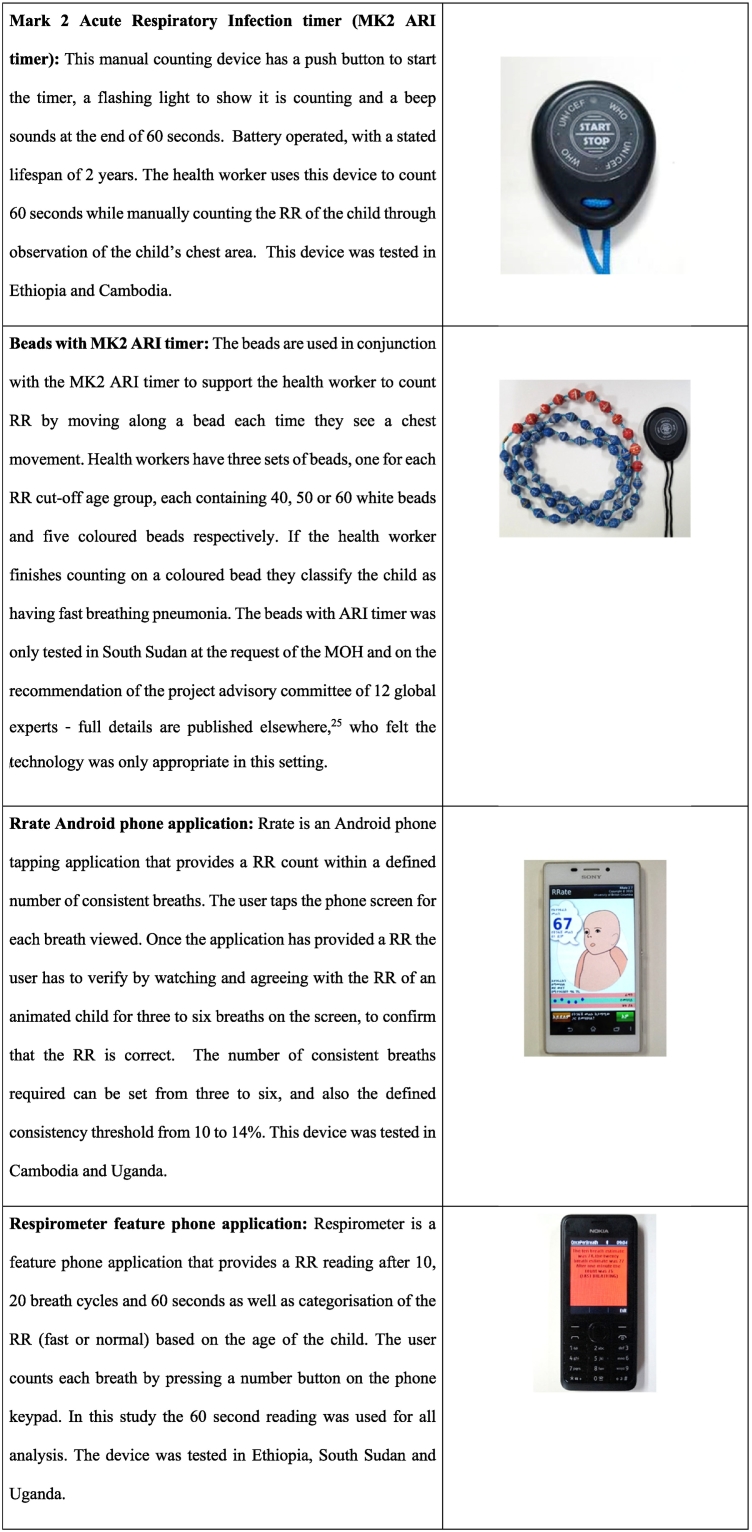
Respiratory rate diagnostic aids evaluated in the study.

This was a prospective, multi-centred, hospital-based, single-blinded, comparative trial of the performance of four RR devices (Fig. 1) when used by CHWs to detect symptoms of pneumonia. The study was conducted from February to June 2015 in district hospitals in four countries: Borkeo Hospital in Cambodia, Yrgalem District Hospital in Ethiopia, Mpigi Health Centre IV in Uganda, and in Aweil General Hospital in South Sudan. All countries had a high proportion of under-five deaths caused by pneumonia (16–21%) [21] and all were implementing Ministry of Health defined iCCM and IMCI programmes [10], [12], [22]. The two RR counters evaluated in each country were selected based on individual country context and formative research [23]. Each device had the same sample size in each country. At a technical consultation on the ‘Evaluation of tools for detecting the symptoms of pneumonia’ [24] a group of 27 global experts agreed that as no gold standard method to establish RR exists, the term ‘reference’ standard should instead be used; the reference standards recommended for the study were 1) an automated RR monitoring device (Masimo Root patient monitoring and connectivity platform with Phasein ISA CO_2_ capnography) that used a nasal cannula to capture CO_2_ to generate a continuous RR measurement and 2) human expert counters standardised to count RR using a stop watch. While both reference standard methods were used in the study, the human expert counters, unlike the automated RR monitoring device, did not count RR simultaneously with the test device, hence these data are not presented in this paper.

The study was approved by ethical review boards in each study country at national or regional level - in Ethiopia from the Southern Nations Nationalities Peoples' Region Health Bureau Health Research Review Committee (Ref: 6-19/10342); in Uganda, from the Uganda National Council for Science and Technology (UNCST) (ref. HS 1585); in South Sudan from the Research and Ethics Committee at the Government of South Sudan, Ministry of Health (Dated 23/05/2014); and in Cambodia from the National Ethics Committee for Health Research (Ref: 0146 NECHR), Ministry of Health, and by the Regional Ethics Committee in Stockholm, Sweden (Ref. 2017/4:10). A scientific advisory committee of 12 global experts in child health approved the protocol.

All children 0 to < 2 m of age and children aged 2 to ≤ 59 months with history of cough and/or difficulty breathing were included in the study once parental consent was given. Exclusion criteria were children with an illness for greater than two weeks or having one or more of the following symptoms: severe dehydration, agitation, inconsolable, neck stiffness, active convulsions/fits, unconscious/lethargic, not breastfeeding and vomiting everything, or children of caregivers less than 18 years of age.

CHWs were trained for two days by master trainers on a refresher of the iCCM classification, referral and treatment algorithms for fast breathing pneumonia, including counting RR and on how to use the two new test devices. All had to achieve a score of 85% in a RR counting competency test before participating in the device evaluation. The research team in each country were all medical officers with at least two years research experience and were trained for five days prior to data collection on research procedures, RR counting and reference standard operating procedures. The team consisted of a project manager, a research officer, two research nurses and two research assistants. The research nurse, who was tasked with attaching the nasal cannula and monitoring the Masimo reference standard, received specific training from a case management specialist on how to operate the device, attach the nasal cannula, and ensure a valid reading was obtained. A pilot test of all elements of the study was conducted in each country in a five-day complete run through with five community health workers. The pilot included the training package, the data collection tools and all standard operating procedures. The pilot was attended by the research team and a debrief session was conducted at the end to review if any amendments were required to any elements of the study before data collection started.

### 2.1 Procedures

All children who presented at the out-patient departments of the study hospitals between 9 am and 5 pm Monday to Friday were potentially eligible for the study, and were approached in the waiting rooms to be screened by a research nurse. Eligible children were brought to the research room where a research assistant explained the study to the caregiver and took informed consent. The CHW randomly selected one of the two test devices they had been trained on. The recruited child was positioned on the caregiver's lap and the nasal cannula was attached by a trained research nurse. Once calm, two RR measurements were obtained by the CHW with the first test device, and the measurements were recorded by research assistants on paper data forms, along with the simultaneous Masimo capnography reference measurements. Then the CHW used the second test device to obtain a further two RR measurements, and again these are recorded by the research assistants, along with the reference standard RR measurements. The research assistants recorded any failed attempts using the test devices and the reasons for this, along with the state of the child during each assessment. The CHWs were also asked to classify the RR into fast or normal breathing after each measurement. The CHWs were blinded to the reference measurements and classifications. Each health worker saw a maximum of six children during the data collection period. Clinical management decisions were made independently by the responsible clinical team at each facility, not taking into account the test device results. All data were collected on paper case report forms and double entered using EpiData version 3.1 (EpiData Association, Odense, Denmark).

### 2.2 Outcomes

The primary outcome was the agreement between the CHW measurements and that of the reference standard, calculated as the mean difference between the CHW observation and the reference standard. We also show, as a secondary outcome, the agreement between the CHW measurements and that of the reference standard, calculated as the proportion of 60s CHW observations with each of the four devices that were ± 2 breaths from the reference standard. As no gold standard exists for respiratory rate measurement we propose to use “reference standard” to define the comparator. As per recommendations by the US Food and Drug Administration [26], when a new test is evaluated by comparison to a non-reference standard, unbiased estimates of sensitivity and specificity cannot directly be calculated. Therefore, the terms sensitivity and specificity are not appropriate to describe the comparative results. Instead, the same numerical calculations can be made, but the estimates are called positive percent agreement (PPA) and negative percent agreement (NPA), rather than sensitivity and specificity. This reflects that the estimates are not of accuracy but of agreement of the new test with the non-reference standard. In addition, quantities such as positive predictive value, negative predictive value, and the positive and negative likelihood ratios cannot be computed since the subjects' condition status (as determined by a reference standard) is unknown. Therefore the secondary outcomes included the agreement in classification of the breath rate into normal or fast, and agreement statistics appropriate for situations when no gold standard exists, such as positive percent agreement (PPA), negative percent agreement (NPA) and Cohen's Kappa statistic. For all of these secondary outcomes the unit of analysis was the child rather than the device measurements.

### 2.3 Statistical Analysis

The sample size calculation was based on the primary outcome, i.e. the precision of the mean difference between the device and the reference standard respiratory count, assuming normal distribution. A standard deviation of SD = 7 for the difference was obtained in a previous study evaluating the performance of RR timers [11], and in requiring a maximal total length of the 95% confidence interval of 4 units, which the same range as the WHO accepted maximal absolute breathing rate deviance (e.g. ± 2 breaths/min), the minimum sample size was 47 children per strata for independent observations. The two age strata in the study were i) 0 to < 2 months and ii) 2 to ≤ 59 months, and one pair of RR devices per country gave a total sample size of 94 children. The sample size was then increased by 50% to n = 141, and rounded off to 150 children per country to accommodate for potential clustering at CHW level [27].

The primary analysis was conducted on the per protocol population, excluding children who were moving or feeding during the RR assessments as per WHO guidelines for counting respiratory rate. To visualise the agreement between different devices and the reference standard, Bland–Altman plots were produced [28]. Bland–Altman plot analysis is a simple way to evaluate a bias between the mean differences, and to estimate an agreement interval, within which 95% of the differences of the test device, compared to the reference, fall. The plots only define the intervals of agreements, they do not say whether those limits are acceptable or not [29]. Proportion agreement was calculated with 95% confidence intervals. Cohen's Kappa statistic (κ) was developed to measure interrater agreement and account for chance [30]. When interpreting Kappa (κ) values Altman recommends agreement at < 0.20 as poor, 0.21–0.40 as fair, 0.41–0.60 as moderate, 0.61–0.80 as good, and 0.81–1 very good [31]. PPA and NPA show the performance of the device being tested in comparison to an existing device using a 2 × 2 table of classifications of fast and normal breathing [32]. Analysis was done using Stata version 13.1 (StataCorp; College Station, TX, USA). Baseline characteristics within each country and strata were summarised using appropriate descriptive statistics. The study protocol is published [33] and is registered with the Australia New Zealand Trials Registry (ANZCTR) (Ref: ACTRN12615000348550). A video detailing the study methods can be seen here (link to: https://www.malariaconsortium.org/resources/video-library/927/protocol-film-implementing-a-trial-to-evaluate-pneumonia-diagnostic-devices).

### 2.4 Role of the Funding Source

The funder of the study had a role in the study conceptualisation and design, but not in the study site selection or data analysis. The corresponding author had full access to all study data and had the final responsibility for the decision to submit for publication.

## 3 Results

**Fig. 2:**
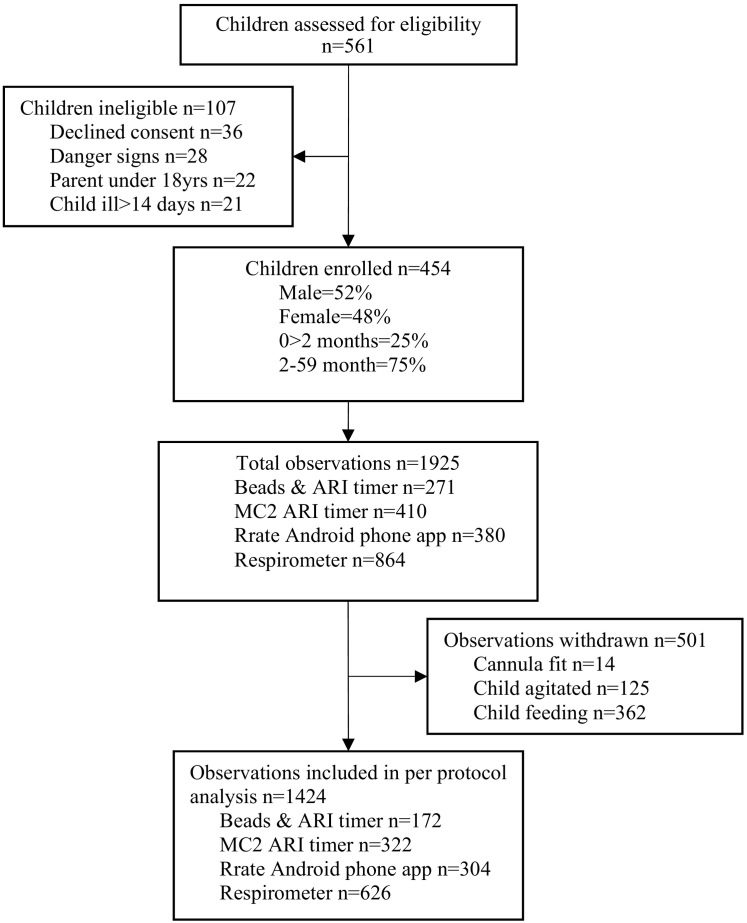
RR timer accuracy evaluation trial profile.

561 potentially eligible children were approached in the study site waiting rooms and assess by a trained research nurse; of which 454 were enrolled; 36 declined consent, 28 had danger signs, 22 had parents younger than 18 years and 21 had been ill for more than 14 days. A total of 1925 RR measurements were recorded, from February until June 2015, across the four countries. In Cambodia and South Sudan, while they did achieve their overall sample, they struggled to recruit children from the younger age group (0 to 2 months). 501 RR measurements were excluded due to protocol violations (agitation or movement, child feeding during assessment or nasal cannula not appropriately attached) (Fig. 2). There were no adverse events reported.

Of the 79 CHWs who took part in the study, 42% were male and 58% were female with a mean age of 32.5 years and had passed a competency assessment after training with a mean score of 89%. The failure rate, i.e. not being able to use the devices to record a RR reading after three attempts was 1.6% (30/1925). Almost all failures (28/30; 93%) were due to the child moving or crying.

All four RR counters had varied mean differences with the reference (Table 1). The MK2 ARI timer (− 0.6 bpm; 95% CI − 2.5 to 1.3) and the Respirometer (− 0.5 bpm; 95% CI − 2.2 to 1.2) had the lowest mean differences overall. The beads with ARI was just at 2 bpm (− 1.9 bpm; 95% CI − 3.8 to − 0.2). The Rrate has the greatest mean difference overall at 5.5 bpm (95% CI 3.2 to 7.8). All four devices had lower agreement with the reference standard in younger children compared with the older children; the Respirometer had the greatest agreement with the reference standard in the youngest children at 1.4 bpm (95% CI − 2.2 to 5.1), and Rrate had the lowest agreement at 10.4 bpm (95% CI 1.2 to 19.5). In the older children MK2 ARI had the greatest agreement with the reference at 0.7 bpm (95% CI − 1.7 to 3.0) and Rrate again had the least agreement at 4.3 (95% CI 2.4 to 6.2).

**Table 1:** The precision, by device, shown by the mean difference of RR measurements from the reference standard.

Devices	Mean difference (95% C.I.)		
	0 to < 2 months	0 to < 2 months	0 to < 2 months
Beads with ARI n = 172	− 4.0 (− 29.4 to 21.4)	− 1.9 (− 3.8 to − 0.1)	− 1.9 (− 3.8 to − 0.2)
MK2 ARI n = 322	− 2.7 (− 5.7 to 0.3)	0.7 (− 1.7 to 3.0)	− 0.6 (− 2.5 to 1.3)
Rrate n = 304	10.4 (1.2 to 19.5)	4.3 (2.4 to 6.2)	5.5 (3.2 to 7.8)
Respirometer n = 626	1.4 (− 2.2 to 5.1)	− 1.3 (− 3.0 to 0.5)	− 0.5 (− 2.2 to 1.2)

All four RR devices had a low level of agreement, with 26–35% of RR measurements being ± 2 bpm from the RR of the reference standard (Table 2). The performance of all RR devices was lower in the young infants (0 to **<** 2 months) compared to the older (2 to ≤ 59 months); however, the study was not powered to compare performance across devices by age group. In the older children (2 to **≤** 59 months) the MK2 ARI and the Rrate devices were in agreement with the reference standard in 40% of the consultations.

**Table 2:** RR measurements by health workers that were ± 2 bpm from the reference standard.

Devices	0 to < 2 months n/N (%)	Agreement ± 2 bpm 2 to ≤ 59 months n/N (%)	Total n/N (%) (95% CI)
Beads with ARI n = 172	*2/4 (50)*^a^	58/168 (35)	60/172 (35) (0.28 to 0.42)
MK2 ARI n = 322	25/125 (20)	79/197 (40)	104/322 (32) (0.27 to 0.37)
Rrate n = 304	5/62 (8)	97/242 (40)	102/304 (34) (0.29 to 0.39)
Respirometer n = 626	34/188 (18)	131/438(30)	164/626 (26) (0.23 to 0.29)

a Small sample size.

The differences in RR counts between CHWs using the four devices and the reference standard, plotted against the average RR of the two techniques, are illustrated by the Bland Altman plots (Fig. 3a, Fig. 3b, Fig. 3c, Fig. 3d). These show that there is a lot of variation in readings for all RR timers particularly in the younger children. For Beads with ARI (Fig. 3a) the plot shows a mean difference of − 1.9 bpm, with limits of agreement (LOA) from − 19.0 bpm to 15.1 bpm, with most variation in CHW readings seen in the older age group. For the MK2 ARI plot (Fig. 3b) the mean difference was − 0.6 bpm, with LOAs from − 25.4 to 23.9 bpm. The plot also shows that for the older children with lower breath rates the device over-counted RR, whereas for older children with higher breath rates the device under-counted RR. For the Rrate (Fig. 3c), the mean difference was 5.5 bpm with wide LOAs ranging from − 24.2 to 35.2 bpm, with more variation in the younger children with higher breath rates. The Respirometer (Fig. 3d) had a mean difference of − 0.5 bpm and the LOAs were wider than the other device, ranging from − 28.6 to 27.5 bpm. Also this device had more variation in the higher breathing rates, with the younger children being over-counted and the older children under-counted when compared to the reference standard.

**Fig. 3a:**
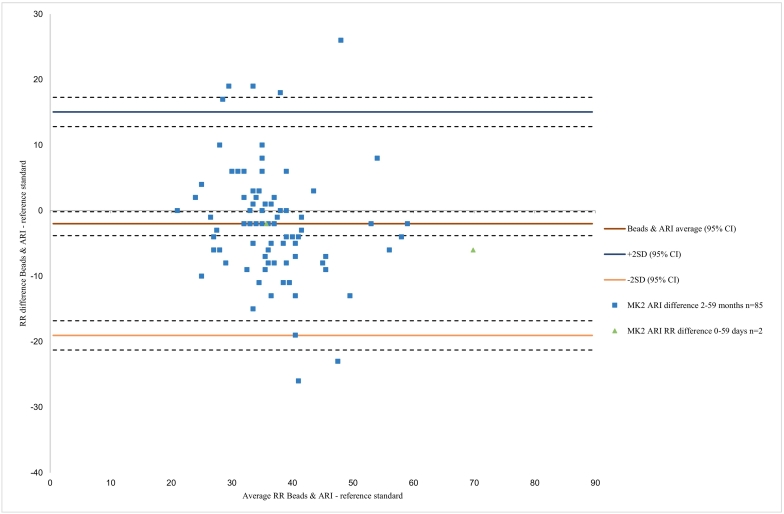
Bland Altman plot for Beads with ARI timer, n = 87.

**Fig. 3b:**
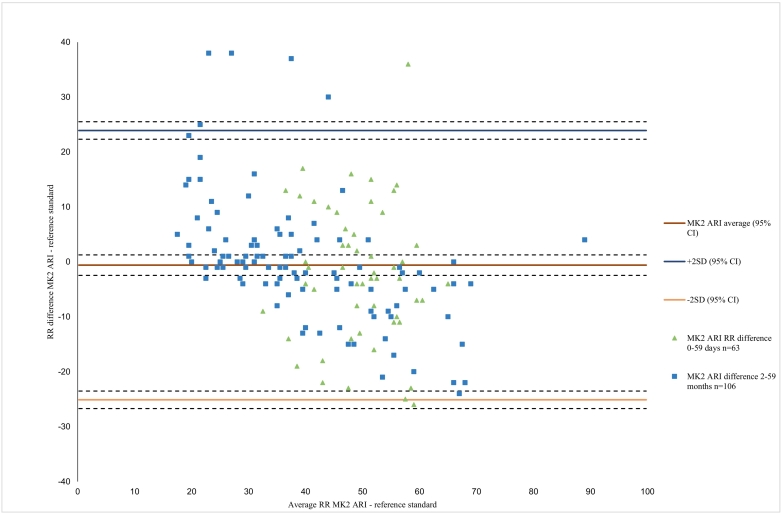
Bland Altman plot for the MK2 ARI timer, n = 169.

**Fig. 3c:**
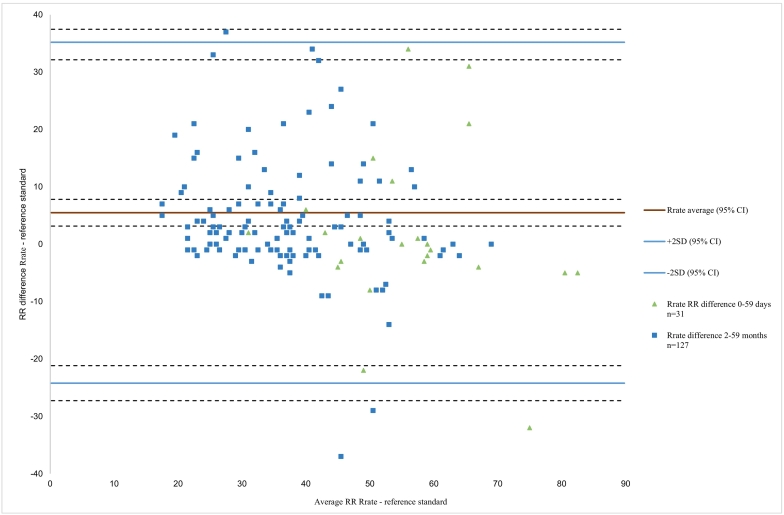
Bland Altman plot for the RRate, n = 158.

**Fig. 3d:**
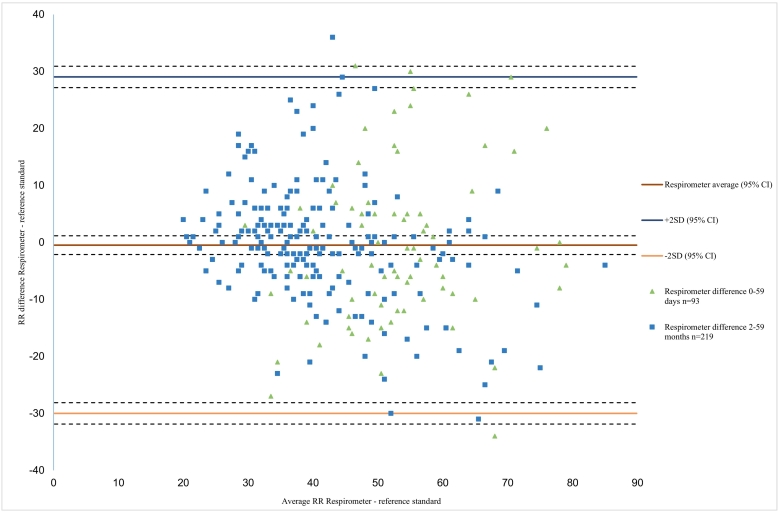
Bland Altman plot for the Respirometer, n = 312.

The agreement of the RR classification into normal or fast breathing for CHWs and reference standard assessments varied widely between the four devices, especially for the young infants. Given the small sample size for beads with ARI for children aged o to < 2 months (n = 4) it is not possible to draw any conclusions on the device performance. The MK2 ARI had the highest κ statistic, both overall (0.49; SE 0.05) and for each of the two age groups, 0.26 (SE 0.08) and 0.62 (SE 0.07) respectively (Table 3).

**Table 3:** Kappa value (κ) for agreement of RR classification into normal or fast breathing, by device and age group.

Device	0 to < 2 months κ (SE)	2 to ≤ 59 months κ (SE)	All κ (SE)
Beads with ARI	*1*.*0 (0*.*5) n* *=* *4*^a^	0.39 (0.07) n = 168	0.41 (0.07) n = 172
MK2 ARI	0.26 (0.08) n = 125	0.62 (0.07) n = 197	0.49 (0.05) n = 322
Rrate	0.13 (0.1) n = 62	0.54 (0.06) n = 242	0.44 (0.06) n = 304
Respirometer	0.19 (0.07) n = 188	0.48 (0.05) n = 438	0.41 (0.04) n = 626

a Small sample.

When comparing the RR classifications of the four devices to the reference standard Rrate had the highest positive percent agreement (PPA) (71%; 95% CI 59.1 to 80.3) and beads with ARI the highest negative percent agreement (NPA) (93%; 95% CI 86.7 to 96.6). In children aged 2 to ≤ 59 months, the Rate again had the highest PPA (73%, 95% CI 60.3 to 83.9) whereas the MK2 ARI had the highest NPA in this age group (97%; 95% CI 91.0 to 99.3) (Table 4). The results in the youngest age group should be interpreted with caution as the sample sizes were small (n = 3–26) and the confidence intervals were very wide.

**Table 4:** Positive percent agreement (PPA) and negative percent agreement (NPA) by device and age strata (95% CI).

Device	0 to < 2 months		2 to ≤ 59 months		Overall	
	NPA	PPA	NPA	NPA	PPA	NPA
Beads with ARI	*100%*^a^ *(15*.*8 to 100*.*0)*	*100%*^a^ *(15*.*8 to 100*.*0)*	41% (27.0 to 56.8)	92% (86.5 to 96.6)	44% (29.5 to 58.8)	93% (86.7 to 96.6)
MK2 ARI	30% (15.6 to 48.7)	90% (82.2 to 95.4)	66% (52.2 to 78.2)	93% (87.3 to 96.5)	53% (41.9 to 63.5)	92% (87.6 to 95.0)
Rrate	61% (35.7 to 82.7)	55% (38.8 to 69.6)	73% (60.3 to 83.9)	84% (77.9 to 89.1)	71% (59.1 to 80.3)	78% (72.4 to 83.5)
Respirometer	36% (22.9 to 50.8)	82% (74.4 to 87.9)	65% (57.2 to 72.3)	83% (77.5 to 86.8)	58% (51.3 to 64.9)	82% (78.3 to 85.9)

Data are percentage (95% CI). Data for positive percent agreement and negative percent agreement were calculated against the reference.

a Small sample.

## 4 Discussion

This study shows that while community health workers (CHWs) using four different RR counting devices are able to obtain respiratory rates (RR) from children in the majority of cases, the agreement of their measurements with the reference standard was low for all devices tested. As in previous studies in Zambia and Uganda, where expert clinicians were used as the reference standard to assess agreement with CHW measurements [8], [34], our study also shows a lot of variability between the CHWs and our automated reference standard RR count. Our data shows that it was especially difficult for CHWs to obtain an accurate count (± 2 breaths) in young infants, in which only 8% to 20% of the assessments were in agreement with the reference standard, regardless of the RR device used. The agreement between the tested devices and the reference standard was significantly higher for older children, ranging from 30 to 40% in the 2 to ≤ 59 month-olds, which is also reflected in a previous study in Zambia where decreased RR variability was seen in older children. There was no significant difference in performance between the four devices tested and, unexpectedly, the three improved manual devices tested in our study (beads with ARI timer, RRate and the Respirometer) all showed lower agreement than studies of completely manual counters, where 46% of observations were ± 2 breaths from the reference in Zambia, and 64% in Uganda [8], [35]. This further affirms our findings that counting RR manually, with breaths being difficult to see and count being hard to maintain without interruptions that require the count to be repeated, is a difficult procedure to do accurately and more is required of a device than simply supporting the health workers to keep count of the number of breaths a patient takes over 60 s. The devices in our evaluation relied on the CHW to observe and manually mark the breaths, and could explain why all devices performed poorly.

Similarly, and likely as a result of low accuracy in RR counting, there were low levels of agreement between the classification of fast and normal breathing compared to the reference standard; for all devices the Cohen's κ statistic was lower than observed in previous studies [4], [34]. However, the MK2 ARI timer in the older age group had positive and negative percent agreement in a range similar to previous studies of ARI timer performance in Zambia, Malawi [36], and Uganda [4]. The marked difference in device performance between age groups of children in this study needs to be considered when developing new diagnostic aids for these settings, in particular for new-borns and young infants where the pneumonia burden is the highest.

For all four devices tested there was a lot of variability in the RR readings, as shown in the Bland Altman plots, with both positive and negative discrepancies observed for all devices. In all devices except the respirometer there was a tendency for more variability in the young infants with higher breathing rates. This could be due to the manual nature of these devices, which make it more difficult to count for higher breath counts, typically seen in younger children. When comparing the mean differences (or bias) in RR readings between the four devices and the reference standard, three of the four devices were within ± 2 bpm, similar to what was observed in another study by Gan et al. [37], and show that these devices usually provide counts slightly lower than the reference standard (− 0.6 to − 1.99). In contrast the RRate device mean difference was positive (5.5 bpm) and significantly greater than what was found in a previous study, where the documented bias when compared against the standardised video reference was 0.6 bpm [37]. Our findings indicate that this device provides RR counts greater than the reference standard, which is not ideal in a pneumonia diagnostic device, as it could lead to over diagnosis and subsequent over treatment with antibiotics. However, under detection of fast breathing could be equally or more harmful, as children with true fast breathing pneumonia would go untreated.

In all devices the limits of agreement were wide, and significantly wider than in previous studies [37], [38]. A possible explanation for this could be that our study was conducted by CHWs who used the devices on sick children in a real-life setting with higher breathing rates, rather than observing videos or healthy children in a controlled setting [39]. Also the reference standard used in our study was different to those used in other studies, where manual counting by experts was often used as a reference. Given the inherent variation in RR that different reference standards generate it would be beneficial to have global consensus on which standardised reference standard or methodology to use for evaluation of future devices [40], [41].

RR in children with cough and/or difficult breathing is still an important predictor of pneumonia in children under five in malaria endemic settings, with sensitivity ranging from 76–81% and specificity from 60–89% [42], [43], [44], [45]. Studies at the community level have shown relatively high sensitivity (75–81%) and specificity (81–83%) of CHW classification of RR in children using the ARI timers demonstrating adequate abilities to count and classify breathing rates in children using WHO guidelines [4], [8], [34]. Due to the shift in the epidemiological context of infections [46], technical advances for prevention and treatment [47], and further evidence becoming available on biomarkers [48], the usefulness of including other clinical signs, such as fever and work of breathing, in the clinical management algorithms for childhood pneumonia is ongoing [46], [49]. Work is also being undertaken to harmonise and redesign the existing WHO guidelines [50]. Use of pulse oximetry screening for detection of severe pneumonia, and host biomarker point of care tests (POCTs) like C-reactive protein (CRP) and procalcitonin (PCT) for detection of bacterial pneumonia, are being investigated [49], [51], [52]. While a study in Tanzania recently showed that the use of POCTs in a modified electronic algorithm resulted in a 49% lower relative risk of clinical failure compared to routine care while reducing antibiotic use, the use of POCTs in primary care is only recommended for higher risk children, to avoid over referral. For example, the positive predictive value of the CRP test to diagnose radiological pneumonia in children with fever and cough drops from 54% to 32% when fast breathing is removed from the algorithm [46], [53]. Hence, respiratory rate counting will continue to play an important role, along with assessing for danger and referral signs, even when POCTs become available in routine care, and the development of improved diagnostics aids for facilitating counting should continue to be a priority until further evidence is presented.

While capnography has been used as a reference standard in another recent study of a similar RR diagnostic aid [54], and while the accuracy of the Masimo reference device has been validated at ± 1 bpm [55], there is currently no data on how well it performs in children less than 59 months, where an infant sized nasal cannula could have provided a better fit. The research teams in each country were specially trained on the optimal use of the device and we have removed from the analysis any of the observations where they reported having issues attaching the nasal cannula on the children. Another limitation is the selection of mean difference as the primary outcome for this study. Given that mean difference does not account for positive and negative readings in the overall measurement it may not always be the most appropriate statistical measure to use when evaluating diagnostic aids. Therefore we have focused the discussion in this paper on other measures of agreement between the CHW measurements and that of the reference standard, such as the proportion of 60s CHW observations with each of the four devices that was ± 2 breaths from the reference standard. This more effectively shows whether the test device is under or over diagnosing fast breathing pneumonia, which is very important in assessing diagnostic performance.

In conclusion, of the four RR devices tested in this study, none performed sufficiently well in the hands of trained CHWs. As the MK2 ARI is the most affordable option, and as most CHWs are familiar with its use, other manual counting devices should not replace the ARI timer. To maximise the effectiveness of community case management of pneumonia, it is recommended that automated, easy to use RR diagnostic aids for assessing symptoms of pneumonia for use in remote, resource poor settings are developed and tested. For this purpose, there is also a need to validate the reference standards available to establish the performance of new devices.

## Contributors

KB participated in the design of the study, supervised the study, participated in the data collection, analysis and interpretation, and drafted and wrote the manuscript. KK reviewed the medical literature, conceived and designed the study, supervised the study, participated in the data analysis and interpretation, drafted and reviewed the manuscript. MP designed the statistical analysis and analysed the data. AM AWS ED TH LM DN MO MP TT AS JN participated in the design of the study and participated in the data collection. MM designed and conducted the training. TA participated in the data interpretation, drafted and reviewed the manuscript. RI participated in the data interpretation. All co-authors reviewed and approved the final version of the manuscript.

## Declaration of Competing Interest

None declared.
